# Milk and Alcohol in Children's Hospitals, etc.

**Published:** 1886-11-06

**Authors:** F. A. Cooper

**Affiliations:** Lady Superintendent. The Victoria Hospital for Children, Chelsea


### MILK AND ALCOHOL IN CHILDREN'S HOSPITALS.

In The Hospital of October 30 you give a table of the value of the consumption of milk and alcohol for the years 1875 and 1885 in various hospitals, this one among others. I wish to draw your attention to the fact that the amounts are not a fair example of the consumption. During the year 1885 this hospital was closed for several months for repairs. I am unable to ascertain the exact time, but it was between four and five months. The average cost of wine for the years 1876-1879 was £39 4*s.*, milk average for same period £200, so that both in milk and wine and spirits the cost is much reduced, and *not* increased, as your table would lead one to suppose.

F. A. Cooper,
Lady Superintendent.

The Victoria Hospital for Children, Chelsea.

